# Proton versus photon deep inspiration breath hold technique in patients with hodgkin lymphoma and mediastinal radiation: A Planning Comparison Of Deep Inspiration Breath Hold Intensity Modulation Radiotherapy And Intensity Modulated Proton Therapy

**DOI:** 10.1186/s13014-018-1066-2

**Published:** 2018-07-03

**Authors:** Christian Baues, Simone Marnitz, Andreas Engert, Wolfgang Baus, Karolina Jablonska, Antonella Fogliata, Andrés Vásquez-Torres, Marta Scorsetti, Luca Cozzi

**Affiliations:** 1 Medical Faculty, Department of Radiation Oncology, CyberKnife Center and Radiation Reference Center of the GHSG, University of Cologne, Kerpener Str. 52, 50937 Köln, Cologne, Germany; 2 Medical Faculty, Department of Hematology and Oncology, University of Cologne, Cologne, Germany; 3 German Hodgkin Study Group, Cologne, Germany; 4 Radiotherapy and Radiosurgery, Humanitas Cancer Center and Research Hospital, Milan, Italy; 5 Department of Biomedical Sciences, Humanitas University, Milan, Italy

**Keywords:** Hodgkin lymphoma, Proton beam therapy, Range uncertainties, VMAT

## Abstract

**Background:**

The benefits of proton therapy in the treatment of patients with Hodgkin lymphoma (HL) are controversially discussed. Therefore we compared intensitiy modulated proton therapy (IMPT) with intensity modulated radiotherapy (IMRT), in the form of volumetric modulated arc therapy (VMAT) in patients with Hodgkin lymphoma (HL), through a comparative treatment planning study.

**Methods:**

Radiation plans for 21 patients with Hodgkin Lymphoma (HL) were computed for IMPT and deep inspiration breath hold (DIBH) VMAT. Plans were optimized and computed assuming deep inspiration breath holding conditions. Dosimetric comparison on standard metrics from dose volume histograms was performed to appraise the relative merits of the two techniques, while proton plan robustness was assessed by re-computing the dose distribution of each plan by varying the Hounsfield Units to stopping power calibration by applying a ± 3 and 4% error.

**Results:**

DIBH-VMAT and IMPT both provided excellent coverage, conformity and heterogeneity of the clinical target volume (CTV) and planning target volume (PTV). IMPT reduced mean doses to the breasts, lungs, heart and normal tissue by 38–83%. IMPT significantly reduced mean doses to the heart to < 5 Gy despite bulky mediastinal disease and decreased breast doses in female patients to < 1 Gy. Despite the simulated 3 and 4% miscalibration errors, no remarkable or measurable impact was observed on the organs at risk (OARs).

**Conclusions:**

This is the first comparison between DIBH-VMAT and IMPT in HL treatment. We could demonstrate statistically significant decreases in all dose/volume metrics of the OARs. Regardless of the planning paradigm used, range uncertainties can substantially under dose the PTV, while perhaps not leading to clinically significant deterioration of CTV coverage. With the geometry applied no impact was observed for OARs, suggesting IMPT as a superior technique for potentially reducing future health risks for HL patients.

## Background

Multi-agent chemotherapy followed by radiation is the standard treatment for patients with Hodgkin Lymphoma (HL). However, even after mediastinal irradiation, follow-up has demonstrated considerable long-term morbidity and mortality caused by cardiovascular disease, or lung and breast cancer [1–7]. Two large randomized studies, the H10 trial of EORTC and the RAPID trial, attempted to eliminate radiotherapy in favorable stage HL, but both failed to meet the predefined inferiority margin when radiotherapy was omitted [8, 9]. Options to decrease toxicity include reducing the radiation dose, the intensity of chemotherapy, the radiation target volume, and inadvertent radiation doses to healthy tissue [10–14]. For HL treatment the chosen target volume has progressively been reduced from total nodal to extended field, then involved field and finally involved node concepts [8, 15]. Furthermore, the International Lymphoma Radiation Oncology Group (ILROG) contributed to standardizing the target volume definition and recommendations for treatment of HL patients, defining the involved site concept as a variation of involved node radiotherapy [11, 12, 16]. Changing the target volume concepts from involved field radiation (IFRT) to involved node (INRT), Weber et al. [17] showed significantly decreased excess relative risk for breast, lung and thyroid regardless of the radiation technique used. Nevertheless, the optimal technique, beam quality and delivery of radiation treatment are the subject of numerous comparative dose-planning studies and are still a matter of debate.

For treatment of mediastinal involvement in HL patients, proton, photon radiation with 3-D conventional techniques versus intensity modulated radiation (IMRT) or volumetric arc techniques (VMAT) have been compared [16–23]. Concerning protons, Hoppe et al. [24] reported about 15 patients who received involved node proton therapy. With a median follow-up of 37 months only two negative events occurred (one relapse and one histology transformation) while the 3-year relapse free survival was 93% and the event free survival was 87%. No severe acute or late toxicity was observed. In a more recent study [25] Hoppe reports the outcome of the treatment of 138 patients. The 3-year relapse free survival rate was 92% for all patients and 96% for the adults (87% for pediatric patients).

Whereas optimized VMAT reduced heart disease, it increased the lung dose and the probability of inducing cancer. Furthermore, results from comparative studies are influenced by dose constraint definitions, different planning goals for various organs at risk (OARs), and heterogeneous anatomical [20], supporting an individualized approach for each patient involving routine planning comparisons [14]. Special beam configurations and patient positioning lead to a significant dose reduction in the volume covered by 20 Gy and 30 Gy (V20, V30) to all critical structures: to the breasts, total lung, heart, left and right ventricle, coronary vessels and to the spinal cord [10, 20]. Moreover, radiation in deep inspiration breath hold (DIBH) reduces the excess risks of myocardial infarction and lung cancer compared with free breathing [23]. Valid clinical data about the reduction of the tumor induction are still missing.

Other publications focused on the comparison of photon techniques versus 3D proton treatment (3D-PRT) [26, 27]. However, no data are available from randomized trials comparing the oncologic outcome for proton radiotherapy (PRT). Data of long-term toxicity are also lacking [4]. The majority of publications only evaluated planning comparisons. Protons deposit most of their radiation dose in tissue near the target volume, depending on the energy. A reduced entrance dose may contribute to a lower dose to healthy tissue. Thus PRT allows low dose sparing with better protection of breast tissue comparing to IMRT/VMAT, which is an important factor to consider when treating Hodgkin lymphomas in female patients [26, 28]. One phase II study [18] on PRT versus 3D proton and IMRT photon radiation for INRT in Stage I-IIIA HL patients demonstrated the lowest mean dose to the heart, lungs, and breasts with PRT, and considerable mean dose reduction for PRT compared to photon IMRT for the heart, lungs and breasts and healthy tissue [19, 21].

The question concerning which parameters (sub)-volumes versus mean dose; high dose volume versus low dose volume is of higher clinical importance is still unanswered and depends on many factors. Compared with passive-beam PRT and conformal 3DRT, helical tomotherapy (HT) achieved better protection of the lungs for doses above 15 Gy. However, mean doses to breasts, lung tissue and heart with PRT were significantly lower compared to 3DRT and HT [28].

A study with 22 patients and a case series have also recently addressed this question. Both came to the conclusion that a further reduction of the burden on risk organs can be achieved through the use of IMPT. Furthermore, the expected reduction in life years can be assumed according to a mathematical model [29, 30]. Our aim was to perform a dose comparative study, using intensity modulated proton (IMPT) and volumetric modulated arc therapy (VMAT) with photons with regard to target volumes and OARs. Additionally, the perturbation of proton dose (PDP), secondary to isocenter calibration errors, was estimated in this planning comparison.

## Methods

### Target volume and OAR delineation

In this single center analysis, all patients with Hodgkin lymphoma and mediastinal involvement were consecutively included in the period from July 2014 to March 2016 and irradiated with a Photon VMAT plan. As part of the study, an IMPT plan was then calculated for each patient and a comparative evaluation was made. Twenty-one patients with a mean age of 34 years (16 women and 5 men) with certain mediastinal involvement of HL in stages I-IV treated at Cologne University Hospital were included in this study (Table 1). The target volumes were delineated according to the guidelines of HL 16/17/18 studies of the German Hodgkin Study Group (GHSG). Regarding the stages delineations were performed following the IFRT or INRT definition in HD 16 and 17. Patients in the HD 18 study received local radiation in PET-positive areas of more than 2.5 cm after chemotherapy. The results in new structures that must be taken into account when setting and subscribing to the target volume. More specifically, the gross tumor volume (GTV) was the lymph node remnant (s) observed on the post-chemotherapy CT. The clinical tumor volume (CTV) was defined as the morphological volume of the initial mediastinal involvement with respect to treatment response and replacement of OAR’s after chemotherapy. CTV included the GTV in patients with partial response after chemotherapy. Adding an 8–10 mm margin in the cranio-caudal axis and 6–8 mm in the lateral axis to the CTV derived the planning tumor volume PTV. The same experienced radiation oncologist delineated the OAR’s. The same target volumes (CTV and PTV) were used for the optimization of the proton and photon plans. All CT scans were acquired in deep inspiration breath hold mode and all the plans were optimized on these datasets.

**Table 1:** Involved lymph node areas of all patients

	Cervical left	Cervical right	Supra/infraclav. Left	Supra/inrfaclav. Right	Upper mediastinum^a^	Lower mediastinum^a^	Right axilla	Left axilla
Pat. 1	–	–	–	–	X	X	–	–
Pat. 2	–	–	–	–	X	X	–	–
Pat. 3	–	–	–	–	X	X	–	–
Pat. 4	–	–	–	–	X	X	–	–
Pat. 5	X	X	X	X	X	–	–	–
Pat. 6	–	–	–	X	X		–	–
Pat. 7	X	X	X	X	X	–	–	–
Pat. 8	–	X	–	X	X	X	–	–
Pat. 9	–	X	X	X	X	X	–	X
Pat. 10	–	–	–	–	X	X	–	–
Pat. 11	–	–	–	–	X	X	–	–
Pat. 12	–	–	X	X	X	–	–	–
Pat. 13	–	–	–	–	X	X	–	–
Pat. 14	X	X	X	X	X	X	–	–
Pat. 15	–	–	X	X	X	–	–	–
Pat. 16	X	X	X	X	X	X	X	X
Pat. 17	–	–	–	–	X	X	–	–
Pat. 18	–	X	X	X	X	X	–	–
Pat. 19	–	X	–	X	X	X	–	–
Pat. 20	–	–	–	–	X	X	–	–
Pat. 21	X	X	X	X	X	–	–	–

^a^The border between the upper and the lower mediastinum was defined by the tracheal bifurcation

### Proton beam planning

Intensity modulated proton plans were optimized to deliver 30 GyE to the PTV.

IMPT plans were created by an experienced physicist (L.C.) using beam spot scanning and intensity modulation realized by means of an inverse optimization process via tuning the spot energy and beam weights simultaneously. The ProBeam proton system (Varian Medical systems, Palo Alto, USA) was used as a source of beam data. The basic workflow for IMPT planning was as follows: calculating the beam line settings, optimizing the spot weights for all field simultaneously, post-processing the spot list (where a deliverable spot scanning sequence was generated), and calculating the final dose via summing all the scanning layers, accounting for differences in tissue properties within the patient. The beam arrangement chosen included three or four fields. For all patients 3 fields were defined as one posterior and two oblique-anterior. For some patients, a fourth anterior field was added to improve target coverage. The specific gantry angles were chosen on a patient per patient basis to minimize the volume of lungs crossed proximally to the target as well as for the other organs at risk. The Bragg peak distribution in depth was achieved using various pencil beam energies (with a nominal range from 50 to 170 MeV) and the weights of individual beams were optimized simultaneously for irradiation fields. The dose calculation for all proton plans was performed on a 2.5 × 2.5 × 2.5 mm^3^ grid. The Eclipse treatment planning system (Varian Medical systems, Palo Alto, USA) v.13.6 was used for this part of the study.

### Volumetric modulated arc therapy planning

VMAT plans were optimized by A. V. and W. B. using the same constraints as for the IMPT planning. The planning CT was acquired with a Toshiba LG 16 row scanner with the patient holding their breath in deep inspiration. The deep inspiration breath hold status during CT acquisition was measured and documented using the Real-time Position Management (RPM v. 1.7, Varian Medical Systems (VMS), Palo Alto, USA).

### Dose comparison study

Dose-volume histograms (DVHs) were calculated for the PTV and organs at risk (OARs): lungs, heart, breasts (for females patients) and spinal cord and healthy tissue defined as the fraction of body included in the CT scan minus the target volumes. For the heart the myocardium was also considered and defined as “heart wall”. We defined it as a new parameter, which might be a better prognostic factor of long-term heart toxicity than commonly used heart mean dose. Dose constraints are shown in Table 2 for the target volumes and in Table 3 for the OARs.

**Table 2:** Summary of target volumes dosimetric analysis from Dose Volume histograms

Parameter	Objective	IMPT	RA	*p* (Wilcoxon)
CTV: 481.3 ± 358.5 [108–1355] cm^3^				
Mean [Gy]	30.0	30.1 ± 0.2	30.6 ± 0.2	n.s.
D_1%_ [Gy]	Minimize	31.5 ± 0.5	32.0 ± 0.5	0.01
D_5%_-D_95%_ [Gy]	Minimize	2.0 ± 0.3	2.5 ± 0.4	< 0.01
D_98%_ [%]	> 28.5Gy (95%)	29.4 ± 0.1	28.8 ± 0.4	0.04
PTV: 881.8 ± 597.0 [215.7–2205.7] cm^3^				
Mean [Gy]	30.0	30.0	30.0	–
D_1%_ [Gy]	Minimize	31.7 ± 0.3	32.0 ± 0.4	0.07
D_5%_-D_95%_ [Gy]	Minimize	2.3 ± 0.3	3.5 ± 0.4	< 0.01
D_95%_ [%]	>27Gy (90%)	28.8 ± 0.3	27.3 ± 0.7	< 0.01

*D*_x%_ dose received by the x% of the volume, *V*_x%_ volume receiving at least x% of the prescribed dose, *CI* ratio between the patient volume receiving at least 90% of the prescribed dose and the volume of the total PTV

**Table 3:** Summary of Organs at risk dosimetric analysis from Dose Volume histograms

Parameter	Objective	IMPT	RA	*p* (wilcoxon)
Spinal cord: 41.6 ± 15.6 cm^3^				
D_1%_ [Gy]	Minimize	15.9 ± 5.6 22%	20.3 ± 5.2	< 0.01
Left breast: 467.5 ± 233.9 cm^3^				
Mean [Gy]	Minimize	0.6 ± 0.9 83%	3.5 ± 2.8	< 0.01
D_1%_ [Gy]	Minimize	6.3 ± 6.6	11.9 ± 5.3	< 0.01
Right breast: 494.6 ± 247.4 cm^3^				
Mean [Gy]	Minimize	0.7 ± 1.7 81%	3.7 ± 3.8	< 0.01
D_1%_ [Gy]	Minimize	4.9 ± 9.2	1.9 ± 7.3	< 0.01
Lungs: 4540.0 ± 1538.3 cm^3^				
Mean [Gy]	<15Gy	4.3 ± 1.8 46%	7.9 ± 3.	< 0.01
D_1%_ [Gy]	Minimize	30.0 ± 0.6	28.7 ± 1.0	< 0.01
V_20Gy_ [%]	< 20%	6.7 ± 3.4	9.8 ± 7.5	< 0.01
V_15Gy_ [%]	Minimize	9.7 ± 5.0	18.2 ± 13.1	< 0.01
V_10Gy_ [%]	Minimize	15.0 ± 7.5	32.9 ± 18.6	< 0.01
V_5Gy_ [%]	Minimize	27.8 ± 11.7	52.7 ± 18.8	< 0.01
Heart 554.8 ± 146.2 cm^3^				
Mean [Gy]	Minimize	4.1 ± 3.9 38%	6.6 ± 4.6	< 0.01
D_1%_ [Gy]	Minimize	26.3 ± 8.9	26.8 ± 8.6	< 0.01
Heart wall 133.6 ± 25.9 cm^3^				
Mean [Gy]	<5Gy	2.9 ± 4.0 40%	4.9 ± 4.7	< 0.01
D_1%_ [Gy]	Minimize	20.8 ± 11.5	21.9 ± 10.9	0.05
Healthy tissue				
Mean [Gy]	Minimize	2.3 ± 1.5 49%	4.5 ± 2.1	< 0.01
V_10Gy_ [%]	Minimize	8.5 ± 6.8 48%	16.3 ± 10.7	< 0.01
CI_90%_	Minimize	1.2 ± 0.1	1.2 ± 0.1	n.s.

*D*_x%_ dose received by the x% of the volume, *V*_x%_ volume receiving at least x% of the prescribed dose, *CI* ratio between the patient volume receiving at least 90% of the prescribed dose and the volume of the total PTV

The DVH were assessed quantitatively using a number of appropriate metrics, which included the mean dose (D_mean_), the dose received by 1% of the PTV/OAR’s volume (D_1%_), D_98%_, D_95%,_ D_5–95%_, as well as a variety of V_xGy_ values. For each parameter analyzed, the mean values ±SD over the 2 patient cohorts were analyzed. In addition, the conformity index (CI_90%_), which measures the degree of radiation conformity and should be ideally equal to 1, if the PTV matches exactly the 90% isodose volume, was assessed.

The Wilcoxon matched-paired signed-rank test was applied to evaluate the level of significance of the observed differences between the dose-volume metrics. The threshold for statistical significance was set at < 0.05. The statistical analysis was performed on the Statistical Package for Social Sciences system (SPSS, Ver.22.0, IBM Corp).

### Proton dose perturbation (PDP) and statistical analyses

PDP analysis was performed to investigate one of the possible dosimetric concerns that could be simulated by the TPS. PDP could in fact result from CT calibration errors of the therapeutic protons. The calibration errors were simulated by varying the Hounsfield Units (HU) to stopping power calibration by applying a ± 3 and ± 4% error in CT numbers [31]. His primarily mimics the proton penetration into the HL patient. The analysis was conducted on all OARs and target volumes to appraise the possible loss in coverage or increase in involvement due to these uncertainties.

## Results

### Dose comparative study

**Fig. 1:**
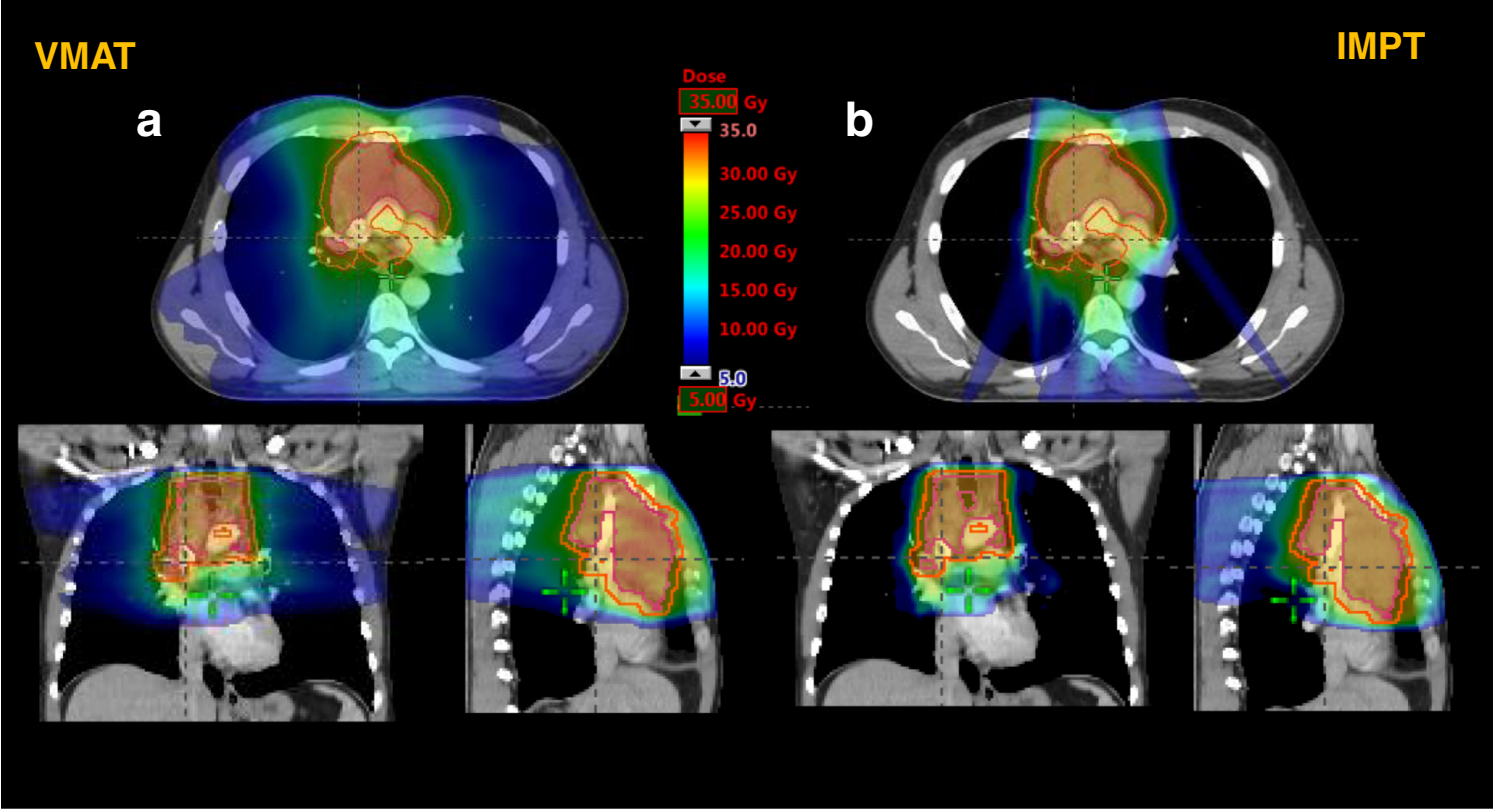
**a** Volumetric arc treatment of a patient with large mediastinal mass with a considerable low does bath (blue). **b** Intensity modulated proton therapy with a significant reduction of low dose bath

Both IMPT and VMAT provided excellent coverage of the planning tumor volume (PTV). No difference was observed with regard to the mean dose to the PTV. Although both techniques fulfilled the planning constraints for CTV and PTV, IMPT provided better conformity, homogeneity and a slightly better coverage. Typical dose distributions in axial, coronal and sagittal planes for one patient and the two techniques are illustrated in Fig. 1. The color-wash display was set to 5–40 Gy with the significantly reduced volume of the low dose bath with IMPT.

The numerical analysis conducted on the dose-volume histograms (DVH) for the various parameters considered are summarized in Table 2 for the targets and in Table 3 for the OARs. The planning strategy adopted for both proton and photon plans, allowed to achieve the level of target coverage required by the study aims. More than 95% of the prescribed dose was in fact computed for the 98% of CTV and more than 90% of the dose was estimated to more than 95% of the PTV. The target homogeneity was expressed by means of D_5%_-D_95%_ and this resulted of the order of 2 Gy for IMPT plans and 2.5 Gy for the photons (relatively to the CTV); both findings demonstrate a very high homogeneity of the dose distributions. Although most of the parameters investigated showed statistically significant differences between IMPT and VMAT, the absolute relevance of these might be clinically minor.

**Fig. 2:**
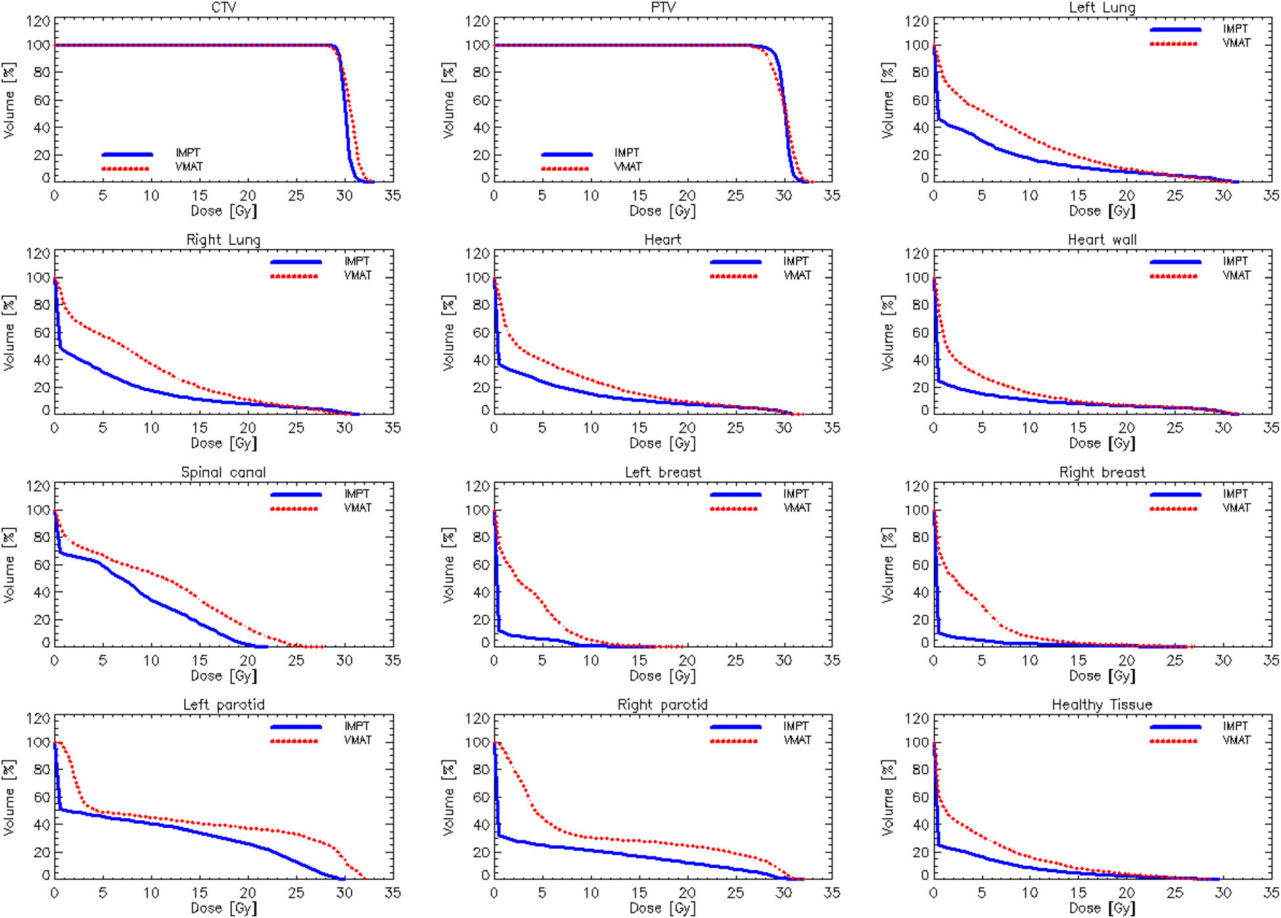
Comparison of dose volume histograms for VMAT (photons, Blue) and IMPT (Red) for CTV, PTV, heart wall, lungs, spinal cord, breast, parotid gland, healthy tissue

Concerning OAR sparing, the data revealed that IMPT led to a statistically significant reduction of mean doses by 38–83% to the lung, the breasts (female patients), the heart and the heart wall (Table 3). IMPT led also to a statistically significant reduction in the near-to-maximum dose in the spinal cord (about 4 Gy of further sparing with IMPT compared to VMAT). Concerning the lungs, all the sub-volumes covered with 5, 10, 15, 20, and 30 Gy could be significantly reduced with IMPT (Table 3 and Fig. 2) while the mean lung dose was nearly halved. Also, the mean doses to the heart and heart wall could be reduced by more than 30%. In particular the average of the mean dose to the entire heart could be reduced to 4 Gy (compared to almost 7 Gy for the photon case). The mean dose and low dose bath (V_10Gy_) to the healthy tissue could be reduced by nearly 50% (Table 3).

### Proton dose perturbation (PDP)

**Fig. 3:**
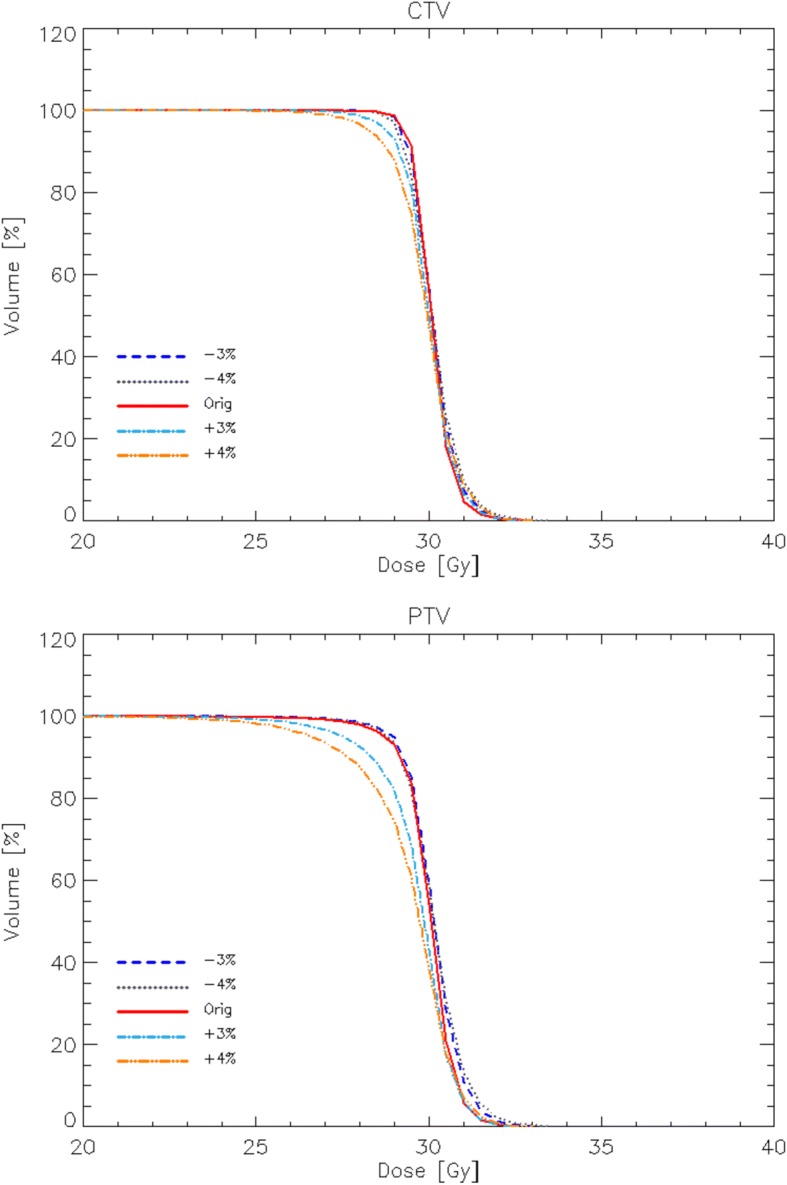
Average DVH for the simulated CTV, PTV and various CT calibration errors

Despite the simulated 3 and 4% miscalibration errors, no remarkable or measurable impact was observed on the organs at risk. In contrast, the effect of range uncertainties was quite relevant for the PTV, although almost completely mitigated for CTV volumes (Table 4). A magnified display of the average DVH for the CTV and PTV and the various CT calibration errors simulated is shown in Fig. 3.

**Table 4:** Summary of target volumes dosimetric analysis from the PDP analysis. Data are reported only for the positive mis-calibrations since these represent the worst scenarios

Parameter	reference	Δ (+ 3%)	Δ (+4%)
CTV			
Mean [Gy]	30.1 ± 0.2	29.9 ± 0.2 -0.7%	29.9 ± 0.3 -0.7%
D_95%_ [Gy]	29.4 ± 0.1	28.9 ± 0.3 -1.7%	28.5 ± 0.4 -3.1%
D_98%_ [Gy]	29.4 ± 0.1	28.4 ± 0.4 -3.4%	27.8 ± 0.6 -5.4%
PTV			
Mean [Gy]	30.0	29.7 ± 0.2 -1.0%	29.4 ± 0.3 -2.0%
D_95%_ [Gy]	28.8 ± 0.3	27.6 ± 0.5 -4.2%	26.7 ± 0.7 -7.3%
D_98%_ [Gy]	28.8 ± 0.3	26.4 ± 0.8 -8.3.%	25.3 ± 1.0 -12.2%

*D*_x%_ dose received by the x% of the volume, *V*_x%_ volume receiving at least x% of the prescribed dose, *CI* ratio between the patient volume receiving at least 90% of the prescribed dose and the volume of the total PTV

## Discussion

This study is one of the first to evaluate intensity-modulated photon (IMRT/VMAT) and intensity modulated proton radiation (IMPT) techniques in deep inspiration breath hold (DIBH) in HL patients with mediastinal involvement. Depending on the stage, we applied the involved field concepts analogous to the HD16 and HD17 study, or the concept of irradiating PET positive remaining lymphoma tissue analogous to the HD18 study [32], including only sites originally involved in the treatment volume. Despite the simulated 3 and 4% miscalibration errors, no remarkable or measurable impact was observed on the organs at risk. We could demonstrate the superiority of IMPT over highly sophisticated photon treatment: Reductions in the mean dose, and absolute or relative volumes of the non-targeted tissues were observed for healthy tissue, lung, and breast tissue. The relative reduction (DIBH-VMAT versus IMPT) in mean body, lung, and breast doses was 38–83%.

A critical issue for all planning comparisons is identifying the organs at risk and prioritizing the sparing of those structures. Our results confirm prior retrospective data, which indicated substantial benefit to selected organs when conventional proton radiation was compared with either conventional 3-D-CRT or conventional IMRT in patients with HL, depending on optimization criteria [18, 19, 21, 24, 26, 28]. Importantly, we could show improvements due to the mean dose to the heart, lung, and breasts in all patients when comparing IMPT against the most comprehensive and sophisticated photon techniques in deep inspiration breath hold. The gantry angles chosen for the plans might play a role as well and, although a class solution was applied, some individualization was applied in the tuning of the angles to minimize the OARs involvement. In some cases, this might have been further improved with completely different approaches but would have also introduced further heterogeneity in the data.

As an example of the adequate planning strategy adopted for IMPT, our findings, both in term of target coverage and organs at risk sparing are consistent and equivalent within the uncertainties with other data from literature, in particular the study from Zeng et al. [33].

Several publications have evaluated the impact of DIBH techniques. Inflating the lungs and pulling the heart downward could reduce doses to the heart and the lungs. Therefore, DIBH can be considered as a standard technique for radiation in patients with mediastinal involvement in HL [10, 23, 34]. The impact of patients’ compliance in the DIBH process might be particularly severe for proton plans. The dosimetric relevance of a sub-optimal DIBH condition was not investigated in this study, due to technical limitations of the tools available and the impossibility to simulate multiple CT scans with different levels of breath hold. It is here acknowledged that, proton delivery systems should be equipped with appropriated breath monitoring system and that the compliance from patients might play a relevant role. To mention also the fact that not all currently existing proton facilities are equipped with volumetric imaging capabilities while the ProScan system, applied for our study, has this functionality.

The benefit from proton techniques depends on the individual anatomy and location of the HL. Here, we selected patients with mediastinal HL involvement. Although patients with involvement in the superior mediastinum benefit less from proton therapy because the volume of heart irradiated is already minimal [19], even in these situations our results show the benefit of IMPT in sparing mean heart and breast doses.

Different cardiac (sub-) structures are used for dose-volume histogram analysis in planning [19, 26]. The origin of the coronary arteries or distinct single vessels is related to many uncertainties with regard to definition and contouring on planning CT and organ movement during treatment. The importance of contouring and localization and its clinical importance has to be questioned and remains to be been validated. Furthermore, the clinical implication of dose reduction to the different cardiac subunits is still unclear.

Nevertheless, the dose to the heart is a great concern in HL patients. Cardiac magnetic resonance imaging (MRI) performed on long-term survivors after radiation demonstrated changes in valvula function or ventricular function in up to 68% of the patients [5]. Tukenova et al. [35] and Mulrooney et al. [36] showed that the risk of death from cardiac disease was significantly higher in patients who received cardiac doses of 5 to 15 Gy. Data from the Childhood Cancer Survivor Study cohort confirmed a correlation between dose and cardiac late effects. The excess relative risk of developing cardiac disease per Gy was 0.49 (95% CI, 0.26–1.3) in patients who had not received anthracycline-containing chemotherapy. Also, smoking status and Body Mass Index influence this risk [37]. For plan optimization we tried to minimize the mean dose to the heart (wall). In our study, the proton technique leads to a statistically significant reduction in the mean doses.

According to the available data [35–37] our planning goal to decrease the mean dose to the heart to < 5 Gy, could only be reached with IMPT, and not with VMAT. Whether the reduction we achieved, i.e. from 4.9 ± 4.7 Gy with photons down to 2.9 ± 4.0 Gy with protons, is of clinical relevance remains unclear. Thus, close follow-up of these patients treated with protons will be important. However, we expect a better long-term result with regard to cardiac complications, although no data is as yet available for risk reduction associated with mean heart doses < 5 Gy.

Radiation-induced secondary malignancies appear to be a major cause of mortality in HL populations. The majority of secondary malignancies have been observed at the radiation field border [38, 39]. Due to the low dose bath, IMRT is likely to double the incidence of secondary malignancies compared to conventional radiotherapy, from about 1 to 1.75% for patients surviving 10 years [40]. As the radiation dose increases, radiation-induced cell death may become dominant over carcinogenic mutations. Protons with their sharp dose gradient should theoretically be able to reduce the risk of secondary cancer. Discussions are ongoing about the shape of the dose–response relationship for higher doses and the risk of secondary malignancies [17]. In a recently published case report Meyer and colleagues showed that proton beam therapy allowed a better target coverage, a better dose homogeneity and conformity to the planning target volume. Furthermore it reduced volume of healthy tissues receiving low doses although it increases weakly volume of tissues receiving high doses [41].

Travis et al. [40] have shown an increased risk of secondary lung cancer after doses as low as 5 Gy. Excess risk after radiotherapy began 5 yrs after treatment and persisted for more than 20 years. For the lungs, our IMPT procedure led to a 50% reduction in nearly all analyzed sub-volumes (V5, V10, V15, V20) compared with photon treatment. With a mean dose reduction to 4.3 ± 1.8 Gy with protons we may have reached uncritical dose levels with regard to the available data.

A large proportion of the second malignancies observed in HL survivors are breast cancers, which occur primarily in women treated for HL < 30 years of age, suggesting a need to prioritize breast dose in these patients [19]. The risk for breast cancer depends on the age at radiation exposition, the volume, and the dose. Childhood cancer survivors treated with lower delivered doses of radiation to a large volume had a high risk of breast cancer compared to patients treated with high doses to smaller volumes [7]. Travis et al. [40] have observed a 3.2-fold increase in breast cancers with a radiation dose of ≥4 Gy compared to HL patients who received lower doses. Interestingly, even DIBH techniques with photons provided a mean dose < 4 Gy in our patients, specifically doses of 3.5 ± 2.8 or 3.7 ± 3.8 Gy were recorded to the left or right breast with VMAT. In contrast to Hoppe et al. [19] with conventional proton planning, in our study IMPT substantially decreased the mean dose even further to 0.6 ± 0.9 Gy to the left and 0.7 ± 1.7 to the right breast (*p* < 0.001).

Naturally uncertainties still remain as regard to secondary cancer after radiation, which will require long-term studies. Moreover, looking at certain DVH parameters does not take into account different factors, e.g. relative biological effectiveness of the radiation, the radiation type (e.g. photons versus protons), details of the beam delivery (e.g. scanned versus scattered proton beams), and the age, gender and other treatments (chemotherapy) of the patient [19].

As a final point of discussion, we would clarify that we opted for a simple comparison between IMPT and VMAT and did not included in the study fixed field IMRT. Indeed, the comparison between VMAT and IMRT was already performed and reported in [17, 35]. In those studies, we did not find any relevant or clinically significant difference which might support the use of IMRT over VMAT. In particular for breast and lungs, the data showed that VMAT could slightly reduce their involvement as compared to IMRT. Concerning the risk of secondary cancer induction, with both linear and not linear models we showed that ERR for VMAT is slightly lower than for IMRT in both involved nodes or involved field settings.

## Conclusion

Even compared with highly sophisticated DIBH VMAT techniques in a very experienced treatment center, IMPT provided superior coverage while reducing the mean doses to the breasts, lungs, heart and cardiac wall. Long-term follow-up is needed to confirm the benefits of IMPT over X-ray techniques in terms of late toxicity and secondary malignancies. Regardless of the planning paradigm used, range uncertainties can substantially under dose the PTV, although this might not lead to clinically significant deterioration of CTV coverage. With the geometry applied here, no impact was observed on organs at risk, indicating that IMPT could play an important role in reducing the risk of inducing secondary malignancies such as breast cancer.

## Abbreviations

3D-PRT 3D: Proton treatment
CI: Conformity index
CT: Computer tomography
CTV: Clinical target volume
DIBH: Deep inspiration breathhold
DVH: Dose volume histogram
EORTC: European Organization for Research and Treatment of Cancer
GHSG: German Hodgkin Study Group
GTV: Gross tumor volume
Gy: Gray
HL: Hodgkin lymphoma
HT: Helical tomotherapy
HU: Hounsfield units
IFRT: Involved field radiotherapy
IMPT: Intensity modulated Proton Therapy
IMRT: Intensity modulated Radiotherapy
INRT: Involved node radiotherapy
MeV: Megaelectron volt
OAR: Organs at risk
PDP: Perturbation proton dose
PRT: Proton treatment
PTV: Planning tumor volume
SPSS: Statistic package for social science system
VMAT: Volumetric modulated Arc Therapy
VMS: Varian medical systems
